# Supplementary material for: Effect of Dosing Interval on Compliance of Osteoporosis Patients on Bisphosphonate Therapy: Observational Study Using Nationwide Insurance Claims Data
Source: J Clin Med. 2021 Sep 24;10(19):4350. doi: 10.3390/jcm10194350 (PMC8509687; doi:10.3390/jcm10194350)
Supplement: Supplementary file 1 [file jcm-10-04350-s001.zip › jcm-1358990-supplementary.pdf]

Journal of
Clinical Medicine

MDPI

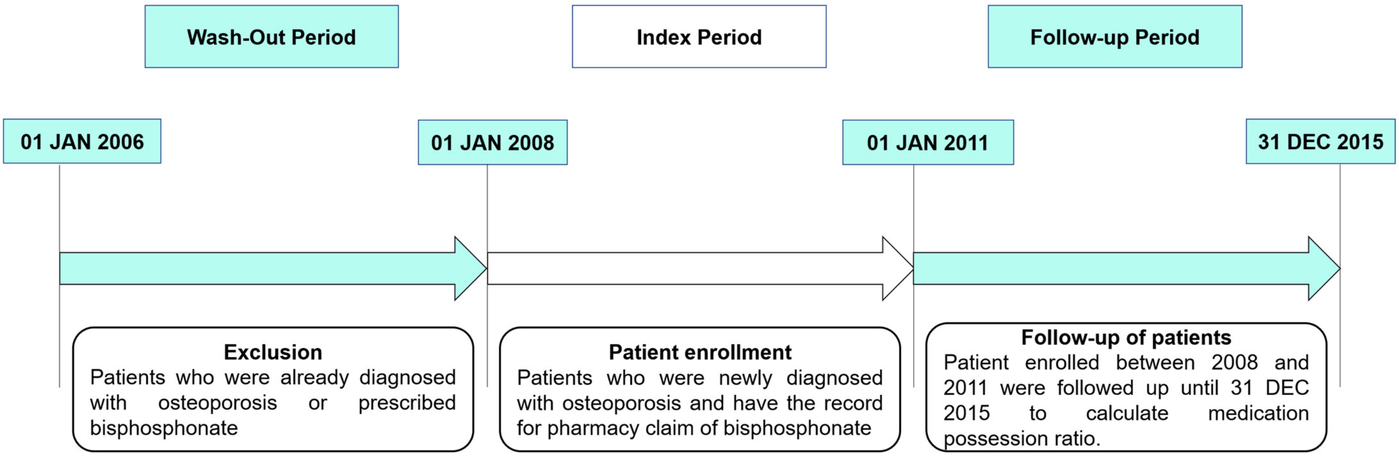

Supplementary Figure S1. Description of study period in extended version: We recruited patients diagnosed with osteoporosis from January 2008 to January 2011. Two years prior to index period (from January 2006 to January 2008) were used as wash-out period. We selected patients who were diagnosed with osteoporosis and prescribed bisphosphonates between 01 January 2008 and 31 December 2010. The first date of bisphosphonate prescription during this period was set as the index date. Only the patients whose data were available until 31 December 2015 were included.

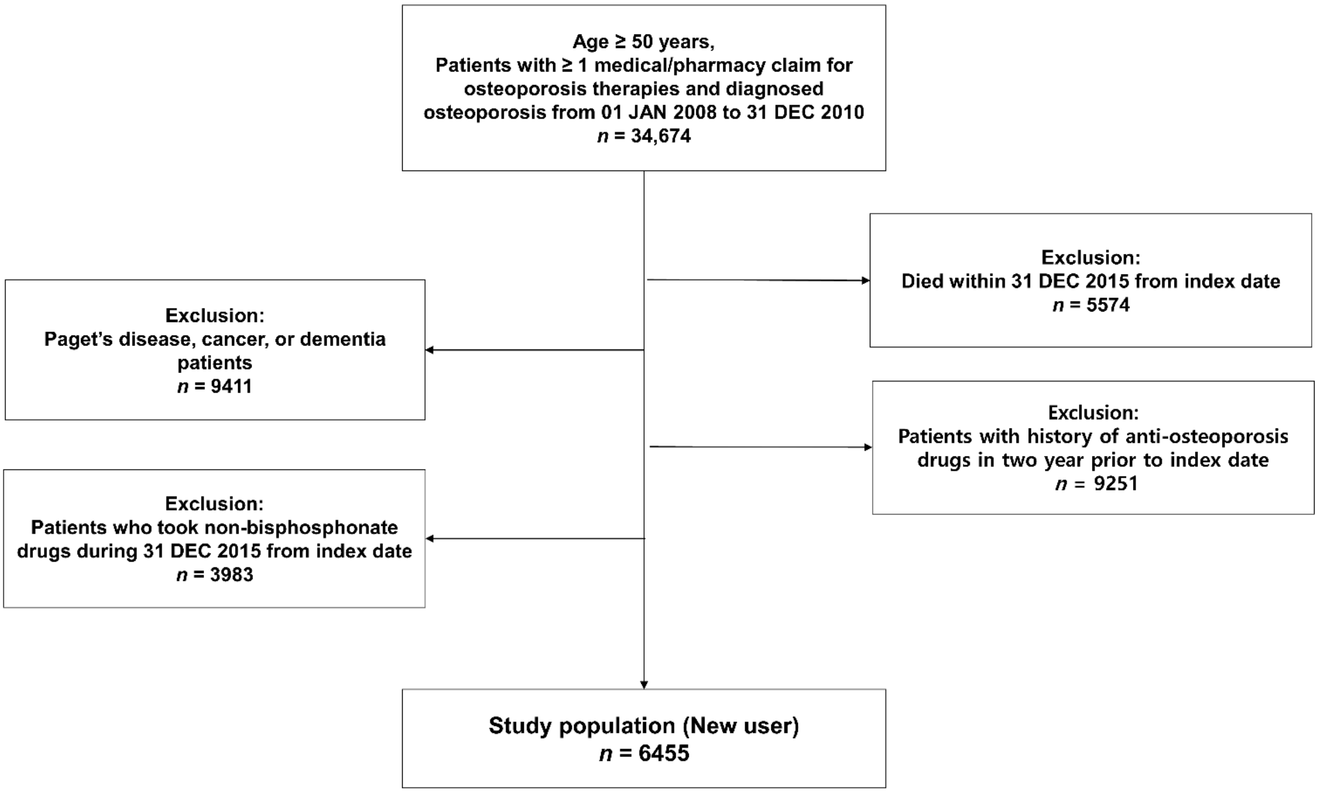

Supplementary Figure S2. Study population of longer-version study: Initial enrollment and exclusion criteria. The first date of bisphosphonate prescription during this period was set as the index-date.

Supplementary Table S1. Types and codes according to dosing interval of bisphosphonate drug.

Dosing Interval	ATC	HIRA General Name Code	Type
Daily	M05BA01	147401ATB	Disodium etidronate 200 mg

	M05BA04	228301ATB	Alendronate sodium 10 mg
	M05BA04	228302ATB	Alendronate sodium 5 mg
	M05BA07	442301ATB	Risedronate sodium 5 mg
	M05BB03	468000ATE	Alendronate sodium 5 mg<br>Calcitriol 0.5μg
Weekly	M05BA04	228303ATB	Alendronate sodium 91.37 mg
	M05BA04	228305ATB	Alendronate sodium trihydrate 91.37 mg
	M05BA07	442302ATB	Risedronate sodium 35 mg
	M05BB03	481100ATB	Alendronate sodium 91.37 mg<br>Cholecalciferol concentrated 26.67 mg
	M05BB03	500200ATB	Alendronate sodium 91.37 mg<br>Cholecalciferol concentrated 53.33 mg
	M05BB07	511200ATB	Risedronate sodium 35 mg<br>Cholecalciferol 56 mg
Monthly	M05BA07	442303ATB	Risedronate sodium 75 mg
	M05BA06	480304ATB	Ibandronate sodium monohydrate 168.75 mg
	M05BB07	518400ATB	Risedronate sodium 2.5hydrate 172.1 mg<br>Cholecalciferol 0.75 mg
	M05BB	523900ATB	Ibandronate sodium monohydrate 168.75 mg<br>Cholecalciferol concentrated granule 240 mg
	M05BA07	442330ATB	Risedronate sodium 150 mg
	M05BA07	500901ATB	Risedronate sodium 150 mg
3 Monthly	M05BA03	207930BIJ	Pamidronate Disodium 15 mg
	M05BA06	480330BIJ	Ibandronate sodium monohydrate 3.375 mg
	M05BA06	641201BIJ	Ibandronate sodium monohydrate 3.375 mg
	M05BA03	207902BIJ	Pamidronate Disodium 15 mg
	M05BA06	480303BIJ	Ibandronate sodium monohydrate 3.375 mg
Yearly	M05BA08	420732BIJ	Zoledronic acid monohydrate 5.33 mg
	M05BA08	420702BIJ	Zoledronic acid monohydrate 5.33 mg

ATC: Anatomical Therapeutic Chemical Classification System; HIRA: Health Insurance and Review Assessment.

Supplementary Table S2. Patient demographic characteristics according to dosing interval of bisphosphonate drug.

		**Total *n* (%)**	**Daily *n* (%)**	**Weekly *n* (%)**	**Monthly *n* (%)**	**3 Monthly *n* (%)**	**Switch *n* (%)**
		281,996 (100)	4367 (1.55)	123,405 (43.76)	40,878 (14.5)	68,575 (24.32)	44,771 (15.88)
**Age, y**	**50–59**	51,850 (100)	745 (1.44)	26,636 (51.37)	7371 (14.22)	10,202 (19.68)	6896 (13.3)
	**60–69**	100,048 (100)	1367 (1.37)	44,597 (44.58)	15,317 (15.31)	22,699 (22.69)	16,068 (16.06)
	**70–79**	90,796 (100)	1407 (1.55)	36,763 (40.49)	12,775 (14.07)	24,164 (26.61)	15,687 (17.28)
	**≥80**	39,302 (100)	848 (2.16)	15,409 (39.21)	5415 (13.78)	11,510 (29.29)	6120 (15.57)
**Gender**	**Men**	23,142 (100)	813 (3.51)	18,952 (81.89)	420 (1.81)	1242 (5.37)	1715 (7.41)
	**Women**	258,854 (100)	3554 (1.37)	104,453 (40.35)	40,458 (15.63)	67,333 (26.01)	43,056 (16.63)
**Area of residence**	**Metropolis**	110,198 (100)	1806 (1.64)	50,717 (46.02)	16,239 (14.74%)	24,738 (22.45)	16,698 (15.15)
	**Cities**	127,923 (100)	1862 (1.46)	55,974 (43.76)	18,254 (14.27)	32,067 (25.07)	19,766 (15.45)
	**Rural area**	43,871 (100)	699 (1.59)	16,711 (38.09)	6385 (14.55)	11,769 (26.83)	8307 (18.94)

**Insurance fee**	**1–5q**	77,547 (100)	1157 (1.49)	33,490 (43.2)	11,114 (14.33)	18,934 (24.42)	12,852 (16.57)
	**6–10q**	42,310 (100)	637 (1.51)	19,029 (44.98)	6138 (14.51)	10,052 (23.76)	6454 (15.25)
	**11–15q**	60,400 (100)	936 (1.55)	26,647 (44.12)	8846 (14.65)	14,587 (24.15)	9384 (15.54)
	**16–20q**	101,739 (100)	1637 (1.61)	44,239 (43.48)	14,780 (14.53)	25,002 (24.57)	16,081 (15.81)
**Fracture history**	**Yes**	30,303 (100)	647 (2.14)	11,940 (39.4)	3610 (11.91)	8701 (28.71)	5405 (17.84)
	**No**	251,693 (100)	3720 (1.48)	111,465 (44.29)	37,268 (14.81)	59,874 (23.79)	39,366 (15.64)
**Facture**	**Forearm**	5571 (100)	89 (1.6)	2236 (40.14)	796 (14.29)	1548 (27.79)	902 (16.19)
	**Hip**	2532 (100)	95 (3.75)	1051 (41.51)	328 (12.95)	701 (27.69)	357 (14.1)
	**Humerus**	896 (100)	18 (2.01)	372 (41.52)	102 (11.38)	260 (29.02)	144 (16.07)
	**Spine**	13,908 (100)	288 (2.07)	5451 (39.19)	1458 (10.48)	4107 (29.53)	2604 (18.72)
	**Others**	2945 (100)	59 (2)	1090 (37.01)	401 (13.62)	802 (27.23)	573 (19.46)
	**Multi fractures**	4471 (100)	98 (2.19)	1740 (38.92)	525 (11.74)	1283 (28.7)	825 (18.45)
**Surgery history**	**Yes**	7865 (100)	209 (2.66)	3306 (42.03)	1026 (13.05)	2070 (26.32)	1254 (15.94)
	**No**	274,131 (100)	4158 (1.52)	120,099 (43.81)	39,852 (14.54)	66,505 (24.26)	43,517 (15.87)
**Surgery**	**Forearm**	2134 (100)	29 (1.36)	877 (41.1)	293 (13.73)	603 (28.26)	332 (15.56)
	**Hip**	2172 (100)	89 (4.1)	986 (45.4)	276 (12.71)	501 (23.07)	320 (14.73)
	**Humerus**	525 (100)	11 (2.1)	210 (40)	75 (14.29)	140 (26.67)	89 (16.95)
	**Spine**	2913 (100)	73 (2.51)	1194 (40.99)	364 (12.5)	793 (27.22)	489 (16.79)
	**Multi fractures**	121 (100)	7 (5.79)	39 (32.23)	18 (14.88)	33 (27.27)	24 (19.83)

Supplementary Table S3. MPR according to dosing interval of bisphosphonate drug and patients' demographic subgroups.

		**Total**		***p*-Value**	**Daily (*n* = 4367)**	**Weekly (*n* = 123,405)**	**Monthly (*n* = 40,878)**	**3 Monthly (*n* = 68,575)**	**Switch (*n* = 44,771)**
		**N (%) [#]**	**Mean (95% CI)**		**Mean (95% CI)**	**Mean (95% CI)**	**Mean (95% CI)**	**Mean (95% CI)**	**Mean (95% CI)**
**Age**	**50–59**	51,850 (18.4)	49.3 (49.0–49.6)	<0.001 *	37.1 (36.8–37.4)	41.3 (41.0–41.6)	47.5 (47.2–47.8)	56.7 (56.4–57.0)	67.9 (67.7–68.1)
	**60–69**	100,048 (35.5)	52.7 (52.5–52.9)		39.8 (39.6–40.0)	42.4 (42.2–42.6)	51.3 (51.1–51.5)	61.0 (60.8–61.2)	68.1 (67.9–68.3)
	**70–79**	90,796 (32.2)	49.5 (49.3–49.7)		37.5 (37.3–37.7)	37.2 (37.0–37.4)	47.1 (46.9–47.3)	57.3 (57.1–57.5)	64.3 (64.1–64.5)
	**≥80**	39,302 (13.9)	42.6 (42.3–42.9)		32.1 (31.8–32.4)	31.0 (30.7–31.3)	39.3 (39.0–39.6)	45.0 (44.7–45.3)	60.1 (59.8–60.4)
**Gender**	**Men**	23,142 (8.2)	38.3 (37.9–38.7)	<0.001 **	36.2 (35.8–36.6)	33.6 (33.2–34.0)	18.9 (18.6–19.2)	39.8 (39.4–40.2)	62.0 (61.6–62.4)
	**Women**	258,854 (91.8)	50.7 (50.6–50.8)		37.3 (37.2–37.4)	40.2 (40.1–40.3)	48.0 (47.9–48.1)	56.7 (56.6–56.8)	65.8 (65.7–65.9)

**Area of residence**	**Metropolis**	110,198 (39.1)	50.4 (50.2–50.6)	<0.001 *	38.3 (38.1–38.5)	40.9 (40.7–41.1)	49.4 (49.2–49.6)	56.7 (56.5–56.9)	66.6 (66.4–66.8)
	**City**	127,927 (45.4)	49.6 (49.4–49.8)		39.7 (39.5–39.9)	39.0 (38.8–39.2)	47.7 (47.5–47.9)	56.5 (56.3–56.7)	65.8 (65.6–66.0)
	**Rural area**	43,871 (15.6)	47.8 (47.5–48.1)		27.1 (26.8–27.4)	34.4 (34.1–34.7)	43.1 (42.8–43.4)	55.4 (55.1–55.7)	63.2 (62.9–63.5)
**Insurance fee**	**1–5q**	77,547 (27.5)	49.6 (49.4–49.8)	<0.001 *	36.3 (36.1–36.5)	39.6 (39.4–39.8)	47.2 (47.0–47.4)	55.6 (55.4–55.8)	65.0 (64.8–65.2)
	**6–10q**	42,310 (15.0)	48.7 (48.4–49.0)		35.9 (35.6–36.2)	38.4 (38.1–38.7)	47.1 (46.8–47.4)	55.6 (55.3–55.9)	65.7 (65.4–66.0)
	**11–15q**	60,400 (21.4)	49.5 (49.2–49.8)		36.8 (36.5–37.1)	39.0 (38.7–39.3)	47.8 (47.5–48.1)	56.5 (56.2–56.8)	66.2 (66.0–66.4)
	**16–20q**	101,739 (36.1)	50.0 (49.8–50.2)		38.3 (38.1–38.5)	39.2 (39.0–39.4)	48.2 (48.0–48.4)	57.2 (57.0–57.4)	65.9 (65.7–66.1)
**Facture history †**	**Yes**	30,303 (10.7)	51.0 (50.6–51.4)	<0.001 **	40.4 (40.0–40.8)	39.9 (39.5–40.3)	49.0 (48.6–49.4)	53.8 (53.4–54.2)	66.1 (65.8–66.4)
	**No**	251,693 (89.3)	49.4 (49.3–49.5)		36.5(36.4–36.6)	39.1 (39.0–39.2)	47.6 (47.5–47.7)	56.8 (56.7–56.9)	65.6 (65.5–65.7)
**Facture †**	**Forearm**	5571 (18.4)	49.7 (48.8-50.6)	<0.001 *	47.1 (46.1–48.1)	37.8 (36.9–38.7)	48.2 (47.3–49.1)	54.4 (53.6–55.2)	65.9 (65.2–66.6)
	**Hip**	2532 (8.4)	50.6 (49.4–51.8)		48.0 (46.7–49.3)	43.2 (41.9–44.5)	49.2 (47.9–50.5)	47.2 (46.0–48.4)	66.8 (65.8–67.8)
	**Humerus**	896 (3.0)	50.5 (48.4–52.6)		40.2 (38.3–42.1)	38.8 (36.7–40.9)	48.9 (46.7–51.1)	51.6 (49.6–53.6)	65.7 (63.9–67.5)
	**Spine**	13,908 (45.9)	50.7 (50.1–51.3)		38.2 (37.6–38.8)	39.0 (38.4–39.6)	47.2 (46.6–47.8)	53.6 (53.1–54.1)	65.8 (65.3–66.3)
	**Others**	2945 (9.7)	55.0 (53.8–56.2)		38.4 (37.1–39.7)	39.0 (37.8–40.2)	47.2 (45.9–48.5)	53.6 (52.4–54.8)	65.8 (64.8–66.8)
	**Multi fractures**	4471 (14.8)	51.7 (50.7–52.7)		34.8 (33.8–35.8)	40.7 (39.7–41.7)	48.8 (47.8–49.8)	54.4 (53.5–55.3)	67.1 (66.3–67.9)
**Surgery history †**	**Yes**	7865 (2.8)	50.8 (50.1–51.5)	0.0016 **	41.1 (40.4–41.8)	41.3 (40.6–42.0)	48.8 (48.1–49.5)	53.1 (52.4–53.8)	67.3 (66.7–67.9)
	**No**	274,131 (97.2)	49.6 (49.5–49.7)		36.9 (36.8–37.0)	39.1 (39.0–39.2)	47.7 (47.6–47.8)	56.5 (56.4–56.6)	65.6 (65.5–65.7)
**Surgery †**	**Forearm**	2134 (27.1)	48.5 (47.1–49.9)	0.0019 *	35.3 (33.8–36.8)	37.9 (36.5–39.3)	47.4 (45.9–48.9)	53.0 (51.6–54.4)	64.1 (62.9–65.3)
	**Hip**	2172 (27.6)	51.3 (50.0–52.6)		46.2 (44.8–47.6)	44.5 (43.1–45.9)	49.1 (47.8–50.4)	46.8 (45.5–48.1)	69.5 (68.4–70.6)
	**Humerus**	525 (6.7)	51.4 (48.6–54.2)		41.4 (38.8–44.0)	39.3 (36.5–42.1)	47.4 (44.5–50.3)	53.9 (51.3–56.5)	69.0 (66.7–71.3)
	**Spine**	2913 (37)	51.8 (50.6–53.0)		37.7 (36.5–38.9)	41.3 (40.1–42.5)	50.4 (49.2–51.6)	56.6 (55.4–57.8)	67.4 (66.4–68.4)
	**Multi fractures**	121 (1.5)	55.0 (49.0–61.0)		33.9(28.7–39.1)	45.7 (39.8–51.6)	37.4 (32.1–42.7)	61.9 (55.7–68.1)	73.5 (68.3–78.7)
**Total**		281,996 (100)	49.6 (49.5–49.7)		37.1 (37.0–37.2)	39.2 (39.1–39.3)	47.7 (47.6–47.8)	56.4 (56.3–56.5)	65.7 (65.6–65.8)

* One-way ANOVA test. ** T-test. # Denominator used to calculate the percentage is fixed as the total number of patients. Percentages are over total in columns. † 'Surgery' and 'Fracture' occurred during wash-out and index period.
